# Convolutional Neural Network Defect Detection Algorithm for Wire Bonding X-ray Images

**DOI:** 10.3390/mi14091737

**Published:** 2023-09-04

**Authors:** Daohua Zhan, Renbin Huang, Kunran Yi, Xiuding Yang, Zhuohao Shi, Ruinan Lin, Jian Lin, Han Wang

**Affiliations:** 1 State Key Laboratory of Precision Electronic Manufacturing Technology and Equipment, Guangzhou 510006, China; zhandaohua@mail2.gdut.edu.cn (D.Z.); hrenbin249@163.com (R.H.); kunranyi@163.com (K.Y.); 2112101016@mail2.gdut.edu.cn (X.Y.); 2112201415@mail2.gdut.edu.cn (Z.S.); 3120000456@mail2.gdut.edu.cn (R.L.); linjian7794@163.com (J.L.); 2 School of Electromechanical Engineering, Guangdong University of Technology, Guangzhou 510006, China

**Keywords:** convolutional neural network, X-ray images, wire bonding defects, YOLO-CSS

## Abstract

To address the challenges of complex backgrounds, small defect sizes, and diverse defect types in defect detection of wire bonding X-ray images, this paper proposes a convolutional-neural-network-based defect detection method called YOLO-CSS. This method designs a novel feature extraction network that effectively captures semantic features from different gradient information. It utilizes a self-adaptive weighted multi-scale feature fusion module called SMA which adaptively weights the contribution of detection results based on different scales of feature maps. Simultaneously, skip connections are employed at the bottleneck of the network to ensure the integrity of feature information. Experimental results demonstrate that on the wire bonding X-ray defect image dataset, the proposed algorithm achieves mAP 0.5 and mAP 0.5–0.95 values of 97.3% and 72.1%, respectively, surpassing the YOLO series algorithms. It also exhibits certain advantages in terms of model size and detection speed, effectively balancing detection accuracy and speed.

## 1. Introduction

Integrated circuits (ICs) are used in almost all electronic products, including smartphones, personal computers, medical devices, automobiles, industrial robots, and more. With the continuous advancement of technology, the manufacturing precision of IC electronic components has been continually improved, transitioning from the micron level to the nanometer level. This advancement has greatly enhanced the performance of IC electronic components but has also posed manufacturing challenges. During the manufacturing process of integrated circuits, defects in ICs can have a negative impact on production costs and actual usage [1]. A significant proportion of defects in IC electronic components arise from the packaging stage. Therefore, in IC manufacturing, it is necessary to properly manage the two crucial subprocesses: packaging and testing [2]. Wire bonding is one of the most important steps in IC packaging, where metal wires are used to connect the chip and the substrate, determining the quality and reliability of power and signal transmission [3]. Various defects can occur during the wire bonding process, including high loop, low loop, interconnection, the wire being missing, and having a broken wire. These defects often lead to poor transmission of electrical signals, affect the normal function of circuits, and reduce the reliability of chips and electronic devices. At the same time, it may also lead to the need for rework or scrapping of substandard chips during the manufacturing process, thereby increasing production costs. After the equipment is put into use, it may become very difficult to find and repair lead issues if maintenance or upgrades are needed. Therefore, it is crucial to detect the quality of IC wire bonding after packaging in order to improve the yield of IC manufacturing.

Due to the increasing demand for high-quality ICs, various sensing technologies have been developed for obtaining wire bonding images for visual inspection. These include Automated Optical Inspection (AOI) [4], X-ray cameras [5,6], Scanning Acoustic Microscopy (SAM) [7], and others. However, most wire bonding image detection is still performed manually by human operators. Manual inspection is slow and cannot keep up with the high productivity of IC chip production lines, failing to meet the growing demands in industrial production. Furthermore, inconsistencies in the visual observation capabilities of human inspectors, visual fatigue resulting from long-term work, and the monotony of repetitive tasks can lead to human errors or omissions during the inspection process. Therefore, with the advent of large-scale IC production, developing an automatic wire bonding defect detection system with high accuracy and fast speed is of great significance.

In the early stages, traditional defect detection methods utilized threshold or edge information, feature extractors, and classifiers to classify and locate defects. Ngan and Kang [8] proposed an algorithm for detecting wires which first generated wire candidate points using a simple one-dimensional gradient threshold technique and then determined the equation of the wire through the Hough transform. Wang et al. [9] presented a wire defect detection method composed of three processes: image preprocessing, connection detection, and defect classification. They eliminated image noise using median filtering, transformed the image into a binary image to extract wire features, and then conducted defect detection and classification. Perng et al. [4] proposed a detection system that combines image processing algorithms and template matching algorithms for identifying wire bonding defects. Additionally, Perng et al. [10] introduced a detection system that combines image processing techniques (thresholding, binary labeling) with wire bonding simulation techniques to automatically verify the accuracy of wire bonding positions. Chen et al. [3] proposed a data-driven framework (DF) consisting of three modules: data preprocessing, feature engineering, and chip classification. This framework is utilized for analyzing IC images obtained through X-ray and detecting wire bonding defects within the images. They employed a thresholding segmentation method to extract chip image blocks from the original images and locate regions of interest (ROIs) containing wires. Next, they employed a wire segmentation algorithm to obtain wire regions. Finally, geometric features were extracted from the segmented wires, and a classifier was employed to determine the types of wire bonding defects. However, due to factors such as the diversity of wire bonding defects and background variations, most traditional image-processing-based defect detection methods struggle to achieve reliable defect segmentation under low contrast conditions, making it difficult to meet the detection requirements in industrial production lines.

Deep-learning-based object detection methods no longer require handcrafted features but instead learn relevant features directly through training. The detection performance improves with increasing amounts of data, leading to successful applications in various fields [11]. Dimitriou et al. [12] introduced a model based on a three-dimensional convolutional neural network capable of identifying defects on wafers. Wu et al. [13] proposed a novel solder joint recognition method based on Mask R-CNN, enabling classification, localization, and segmentation of solder joint defects. Shu et al. [14] introduced a parallel deep convolutional model for surface quality inspection of LED chips, utilizing softmax regression for defect classification. Wang et al. [15] proposed an object detection network that combines the spatial attention module with YOLOv4 for detecting minute defects. They also constructed a dataset for chip surface defect detection and achieved promising results. Stern et al. [16] employed fully convolutional networks for chip defect detection. Huang et al. [17] proposed a small object detection method based on YOLOv4 to improve the detection performance of small objects. Dong et al. [18] introduced pyramid feature fusion and global contextual attention networks for pixel-level defect detection. Wang et al. [19] presented an improved convolutional neural network (CNN)-based Visual Difference Sensitivity Residual (VDSR) algorithm and a CNN-based classification model for chip solder joint detection. While deep-learning-based object detection methods have made significant progress in detecting and classifying various real-world defect data, meeting the requirements of high accuracy, high speed, and multi-class detection in practical industrial production still poses certain challenges.

To meet the requirements of industrial defect detection, this paper proposes an object detection network model for wire bonding X-ray image defect detection. It achieves accurate detection and localization of defects, fulfilling the demands of high-speed and high-precision visual automated inspection. We combine the adaptive weighted multi-scale feature fusion module SMA proposed in this paper with the network to enhance the attention to different feature scales. Simultaneously, the CBAM (Convolutional Block Attention Module) spatial and channel attention mechanism is introduced to enhance the network’s attention to spatial and channel dimensions, thereby improving the network’s feature extraction capability.

The main contributions of this paper are as follows:

- Based on the C2F module in YOLOv8, we propose a module (C2SF) that can fully extract semantic information at different gradients and construct a feature extraction backbone network with this module.
- We propose a self-adaptive weighted multi-scale feature fusion module (SMA) with different dilation rates to expand the receptive field to predict multi-scale defects. In the SMA module, different weights are given based on the contribution of feature information at different scales to the detection results, thereby making full use of the feature information.
- To meet the requirements of wire bonding defect detection, we designed a new defect detection network framework YOLO-CSS based on the C2SF module and the SMA module. This network significantly improves detection accuracy and speed while maintaining detection speed.

## 2. Related Works

### 2.1. Image Data Acquisition

**Figure 1:**
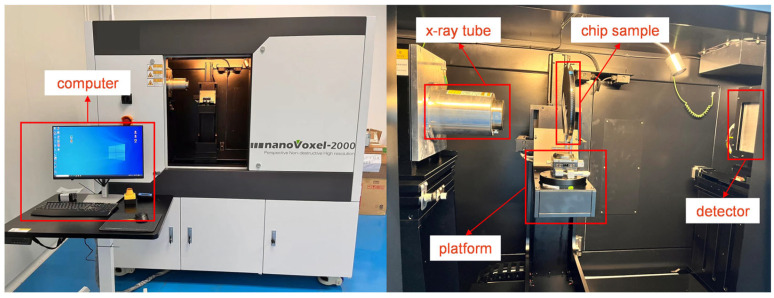
X-ray Image Data Acquisition Equipment.

To ensure the integrity of the chip, the use of non-destructive testing technology is essential. X-ray is a mainstream technology in industrial non-destructive testing. The basic principle is to use X-rays to penetrate the object under test, where the X-rays interact with the atoms inside the object and undergo attenuation, thereby obtaining images of the interior of the object. In simple terms, the larger the atomic number of the atoms that make up the object, the greater the attenuation of the X-rays and the weaker the penetration power, and vice versa [20]. The metal leads inside the chip can cause greater attenuation of X-ray intensity compared to other parts of the material, so a two-dimensional image of the interior of the chip can be reconstructed based on the different intensities of the X-rays. The data for this experiment were collected using a nanoVoxel-2000 X-ray device from Tianjin Sanying to obtain the X-ray images inside the chip, as shown in Figure 1. This device consists of two main parts: data acquisition and image reconstruction. The data acquisition mainly includes the X-ray source, chip sample, rotating platform, and detection panel. The image reconstruction part is mainly completed by a 64-bit computer equipped with a GTX4060 graphics card.

### 2.2. Description of the Dataset

**Figure 2:**
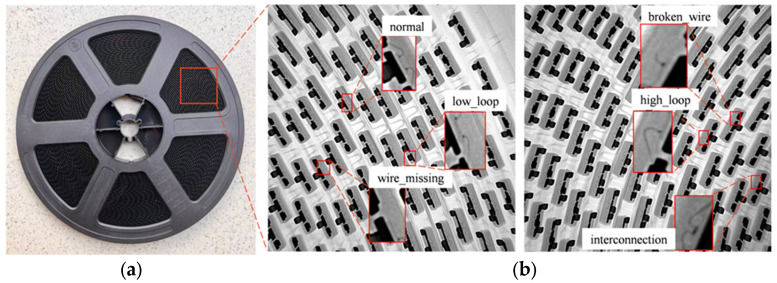
(**a**) The detection target; (**b**) Five different types of defects.

In this paper, we used an X-ray device to collect five common types of defects in the wire bonding process, including high loop, low loop, interconnection, wire missing, and broken wire. As shown in Figure 2, these defects take up a small proportion of the total image area. Actual field measurements show that the minimum enclosing rectangle area for each wire bonding defect is only around 1.5 mm^2^.

In this experiment, 1264 X-ray images of size 1004×1004 pixels with bonding defects were obtained on-site. To enhance the generalization ability of the algorithm, we augmented these defect images through rotation, translation, scaling, adding random noise, changing brightness, and other various random combination methods. In the end, we obtained 3214 data images, each of which contains various types of defects. There are 1324 images with high loop, 1302 images with low loop, 1086 images with interconnection, 1006 images with a broken wire, and 1144 images with a missing wire. Finally, we used the labelImg data annotation software to label the entire dataset and designated 80% of it as the training dataset, with the remaining 20% used as the validation set. All detection algorithms mentioned in this paper were tested based on this dataset.

## 3. Method

### 3.1. YOLO-CSS Framework

**Figure 3:**
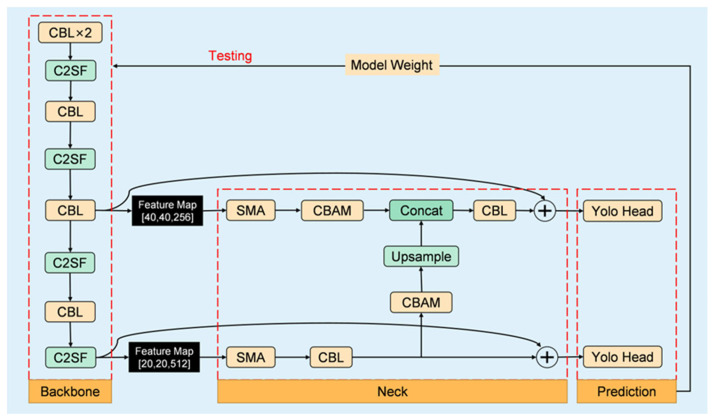
The structure of the proposed method.

Since the advent of the single-stage network represented by the YOLO [21] series, it has developed to its eighth version (YOLOv8). The YOLO series network, with its characteristics of fast detection speed and high detection accuracy, is widely used in industrial detection. However, for the chip lead defects to be detected in this article, YOLO series networks often fail to achieve satisfactory results in terms of speed, accuracy, and model size simultaneously. The YOLO-CSS algorithm proposed in this paper re-designs the network structure of the model based on the official YOLOv5 code. It builds the feature extraction backbone network of YOLO-CSS with the C2SF module as the basic feature extraction module; it also combines the Convolutional Block Attention Module (CBAM) and the self-adaptive multi-scale feature fusion module (SMA) to achieve rapid detection of wire bonding defects. In addition, we also introduced a “skip connection” structure to ensure the integrity of feature information. The YOLO-CSS network structure is divided into three parts: backbone, neck, and prediction. The detailed network structure is shown in Figure 3. In the backbone, the CBL (Conv2d + BN + LeakyReLU) module and the C2SF module are alternately stacked to increase the network depth to extract high-level semantic information. Firstly, the resolution of all input images is uniformly adjusted to 640×640, and two CBL modules with a stride of 2 are used to compress the training images in width and height, while the number of channels is expanded to 64. Next, two C2SF modules and a CBL module are alternately stacked to obtain the first effective feature layer with the size of 40×40×256. Subsequently, C2SF–CBL–C2SF is continued to obtain the second effective feature layer with the size of 20×20×256. These two effective feature layers are passed to the neck part. In the neck, the CBAM attention mechanism and SMA multi-scale feature fusion module are embedded. The CBAM module [22] assigns different weights to feature channels and feature spaces in a learned manner, allowing for different attention for different information and channels and effectively utilizing the features; the SMA module also assigns different weights to feature layers of different scales in a learned manner. Finally, the addition of “skip connections” ensures the integrity of information and alleviates the problem of network degradation to a certain extent.

### 3.2. Backbone Structure of YOLO-CSS

The quality of network model detection results largely depends on the amount of valid information that the feature extraction network can extract. Therefore, the performance of the feature extraction network is very important for the detection results. Inspired by the C2F module in YOLOv8, we designed the C2SF feature extraction module. The difference between C2SF and C2F is that the SE attention mechanism is added in C2SF, which improves the feature extraction performance of the module, and the bottleneck module is replaced with the CMblock module proposed in this paper. C2SF module can obtain rich gradient information. Shallow gradient information can better capture low-level semantic information (such as edge information of leads), while deep gradient information can obtain higher-level semantic information (such as defect types of leads).

**Figure 4:**
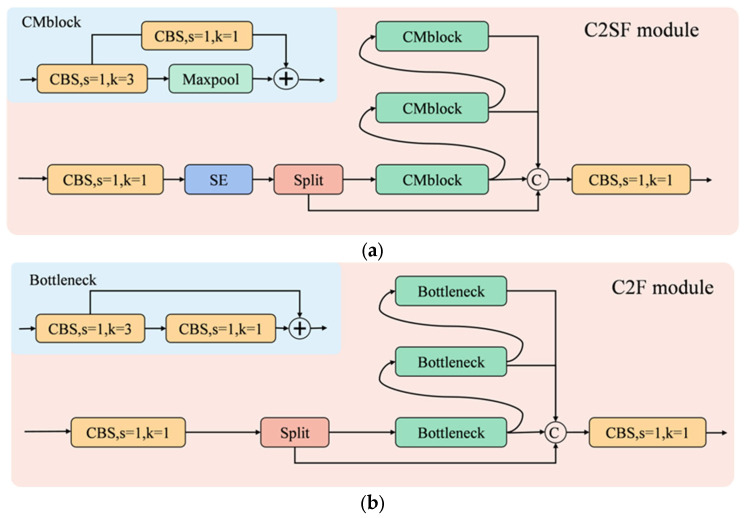
(**a**) Structure of the C2SF module; (**b**) Structure of the C2F module.

The C2SF module is shown in Figure 4a. CBS is composed of ordinary convolution, BN (Batch Normalization), and a SiLU activation function. BN adjusts the data to between 0 and 1 for easier computation, effectively preventing model overfitting and accelerating network convergence; the non-linear activation function enhances the generalization of the network. The CMblock module consists of two CBS modules and a max pooling layer. The process of C2SF’s feature handling is as follows: First, the input features go through a CBS module with a convolutional kernel of 1, and the SE attention mechanism allocates weights to the feature channels. Then, the resulting features are divided into two parts, one of which is passed through the CMblock module to acquire rich gradient information, enhancing the network’s learning capability. The remaining part and the gradient-combined part are then stacked along the channel dimension to prevent information loss. Finally, the CBS module is used to adjust the number of channels to match the input channel count.

### 3.3. Neck Network Based on Weighted Multi-Scale Feature Fusion

**Figure 5:**
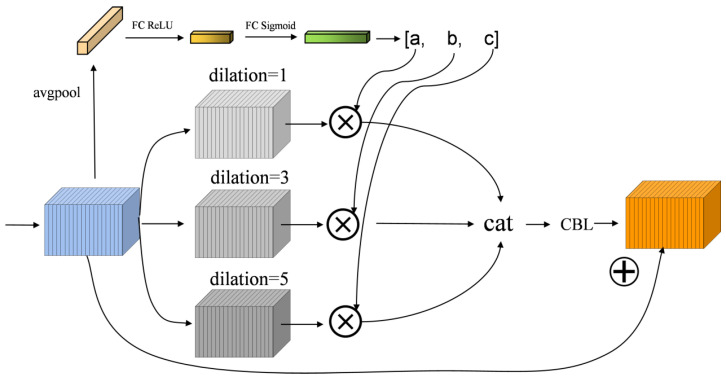
SMA Structure Diagram.

To address the issue of assigning equal weights to different scales in the multi-scale feature fusion part, this paper proposes an adaptive weighting module (SMA), as shown in Figure 5. Adaptive weighting is performed based on the contribution of feature maps at different scales to the detection results in order to suppress non-important feature information. This module performs two operations on the input features.

First, the input undergoes feature extraction through three sets of 3 × 3 convolutional kernels with dilation rates of 1, 3, and 5, respectively, using the dilated-group convolution. To ensure that the size after convolution remains the same as the original input, padding is set to the same value as the dilation rate. The parameter count of the grouped convolution is only 1/g of regular convolution, where g is the number of groups in the grouped convolution. This greatly reduces the network’s parameter count and training time. In object detection tasks, the sizes of the targets to be detected vary. Therefore, using dilated convolutions to obtain feature maps of different scales can be more advantageous for detection, as shown in Figure 5. The three convolutional kernels used in this paper have dilation rates of 1, 3, and 5, respectively. Different dilation rates correspond to different receptive fields that the convolutions can capture.

In the second part, the input feature map of size w×h×c undergoes global average pooling, resulting in a feature vector of size 1×1×c. Then, the obtained feature vector is fed into a fully connected layer to obtain a feature vector of size 1×1×c/reduction, where reduction is the ratio between the number of input channels and the number of output channels in the fully connected layer. Subsequently, the ReLU activation function is applied to enhance the non-linear expressive power of the fully connected layer. The 1×1×c/reduction feature vector is then transformed through another fully connected layer into a size of 1×1×3. Finally, the Sigmoid activation function is used to map the three obtained values to the range [0, 1]. The three obtained values, a, b, and c, are the weight values of the feature maps generated by the three sets of dilated-group convolutions in the first step. The results from the first and second parts are multiplied together and stacked along the channel dimension. Finally, a CBL module with a 1×1 convolutional kernel is used to reduce the number of channels to c, making it compatible for the add operation with the feature map from the original input to avoid the gradient explosion and vanishing problems.

In addition to the SMA module, we also introduced the CBAM spatial-channel attention module in the neck part to further enhance the feature information. To ensure network stability, we introduced “skip connections” before the feature map detection to ensure the integrity of information and partially address the problem of network degradation.

## 4. Experiments

### 4.1. Model Training Details

The experiment was conducted on the PyTorch 1.8.1 deep learning framework with Python version 3.8. The experiment was implemented on a system with an NVIDIA GeForce RTX 4090 GPU with 24 GB of VRAM and an Intel 3.0 GHz i9-13900KF CPU. The batch size was set to 32, and the training was performed for 500 epochs. The image size was uniformly adjusted to 640×640 pixels. The learning rate was set to 0.01, the momentum was set to 0.937, and the weight decay coefficient was set to 0.0005. The dataset was divided into a training set and a test set in a ratio of 80% and 20%, respectively. The training set was used to train the network parameters to minimize the loss function. The test set was used to evaluate the accuracy of the trained network in recognizing wire bonding defects.

### 4.2. The Validation of the Effectiveness of the SMA Module

**Figure 6:**
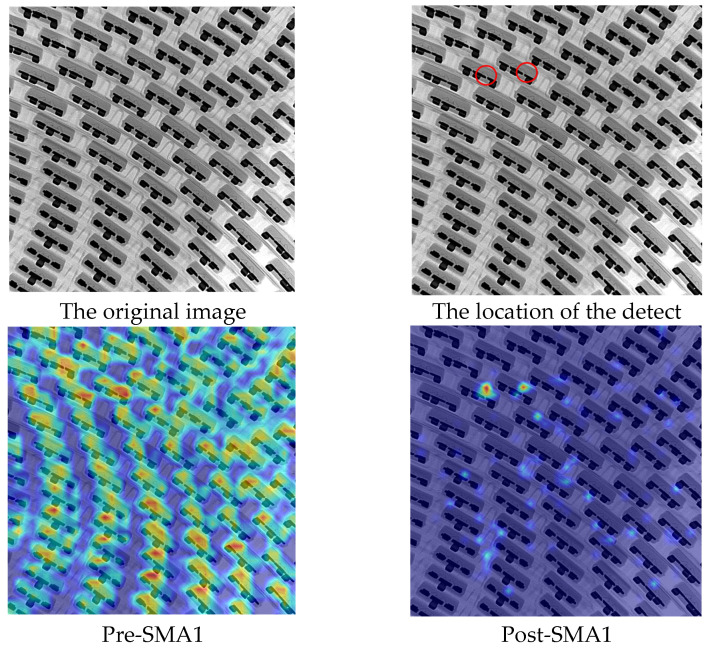

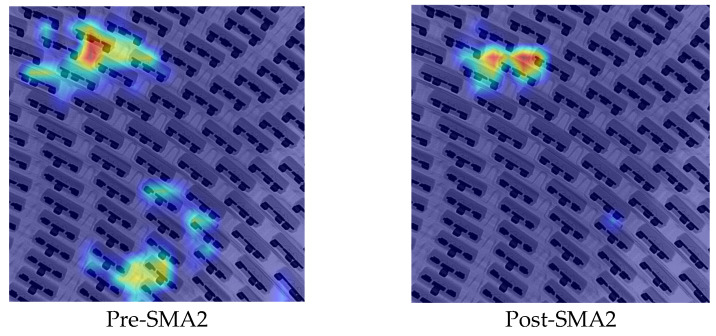
Visualization of feature maps before and after the SMA module.

To validate the effectiveness of the proposed SMA module, we utilized the Grad-CAM algorithm [23] to visualize the network after training the YOLO-CSS model for 500 epochs. The results are shown in Figure 6. A random X-ray image with wire bonding defects was selected, and the feature maps before and after the two SMA modules in the network were visualized for comparison. In this case, SMA1 represents the 40×40×256 feature map. Before entering the SMA1 module, the network’s feature extraction was chaotic and did not focus on the defect locations. After passing through the SMA1 module, the weights of features in other non-defect regions are suppressed due to adaptive weighting, while the weights of the defect locations are enhanced. Before entering the second SMA module (SMA2), the network has a deeper depth, allowing it to express higher-level semantic information. Therefore, the feature visualization results here are much better than those before entering the SMA1 module. After going through the SMA2 module, the network’s focus area can correctly identify the wire bonding defect locations. From this, we can conclude that the proposed SMA module in this paper has excellent feature extraction capabilities and can adaptively adjust the weights of different scales.

### 4.3. Model Evaluation Metrics

In pin defect object detection tasks, Intersection over Union (IOU) is used to determine whether the detected object is a true defect. If the IOU value exceeds a predefined threshold, it is considered a correct prediction; otherwise, it is considered an incorrect prediction. This paper adopts mean Average Precision (*mAP*), Frames Per Second (*FPS*), precision, and recall as evaluation metrics to assess the performance of the algorithm. mAP represents the average *AP* value across all defect categories, where *AP* is the area under the precision–recall curve. In object detection tasks, a higher *mAP* value indicates better overall detection performance of the model across all categories. It is defined as follows:

(1) $$Precision=\frac{TP}{\left(TP+FP\right)}$$

(2) $$recall=\frac{TP}{\left(TP+FN\right)}$$

(3) $$AP=\int_{0}^{1}p\left(r\right)dr$$

(4) $$mAP=\frac{1}{N}\overset{N}{\underset{i=1}{\sum }}AP_{i}$$

*TP* (True Positive) refers to the number of correctly detected defects. *FP* (False Positive) is the number of defects erroneously detected which are actually not defects. *FN* (False Negative) represents the number of missed actual defects. TN (True Negative) represents the number of correctly predicted negative samples. The determination of whether a prediction result is *TP* or *FP* is based on the Intersection over Union (IOU) between the predicted bounding box and the ground truth bounding box. In this experiment, the IOU threshold was set to 0.5. Here, N represents the number of classes, which is five in this paper. FPS (Frames Per Second) refers to the number of images that the target network can detect per second and is commonly used to measure the inference speed of the model. When the threshold of IOU is set to 0.5, the mean average precision (*mAP*) is represented by mAP 0.5. Similarly, mAP 0.5–0.95 refers to the average value of *mAP* under different IOU values when the IOU value increases from 0.5 in increments of 0.05 to 0.95.

### 4.4. Ablation Experiment

To validate the superior performance of CBAM in this algorithm, we selected four commonly used attention mechanisms to replace the attention module in YOLO-CSS. These include Squeeze-and-Excitation Network (SENet) [24], Efficient Channel Attention Network (ECANet) [25], Coordinated Attention (CA) [26], and the Convolutional Block Attention Module (CBAM) used in this paper. As shown in Table 1, the use of attention networks consistently outperformed the non-attention counterpart across various performance metrics. Although the FPS without using attention networks is better than the others, the detection speed with attention networks is still around 130 FPS, which is sufficient to meet the detection requirements. Therefore, we prioritize the use of CBAM attention, which offers better detection accuracy.

### 4.5. Comparison of Results from Different Attention Mechanisms

To further validate the detection performance of YOLO-CSS, we evaluated the algorithm using metrics such as mAP 0.5, mAP 0.5–0.95, precision, recall, and FPS. We compared the performance of YOLO-CSS with several algorithms listed in Table 2. From the detection results in Table 2, it can be observed that our method outperforms other algorithms by 11.6%, 15.8%, 44.7%, 21.4%, 19.6%, 17.5%, 14.1%, 7.3%, 38.3%, and 34.5% in terms of mAP 0.5 performance metric, respectively. In addition to outperforming other algorithms in mAP 0.5, our algorithm also shows significant improvement in mAP 0.5–0.95 compared to other methods. In terms of precision and recall performance metrics, our method achieves a 5.1% and 6.5% improvement over the best-performing YOLOv5 algorithm, respectively. Although the fastest algorithm among the tested methods, YOLOv5, achieves a detection speed of 134.1 FPS, which is 8.2 FPS higher than our method, the overall performance of our method is still superior to YOLOv5. Furthermore, the detection speed of YOLO-CSS is more than sufficient to meet the practical requirements. Additionally, PicoDet [27] has a model size of only 2.98 MB, much smaller than other detection algorithms. However, its detection accuracy is significantly lower than that of YOLO-CSS. In the context of detection, detection accuracy and recall are more important, while the model size often serves as an auxiliary indicator to assess the model’s quality.

**Figure 7:**
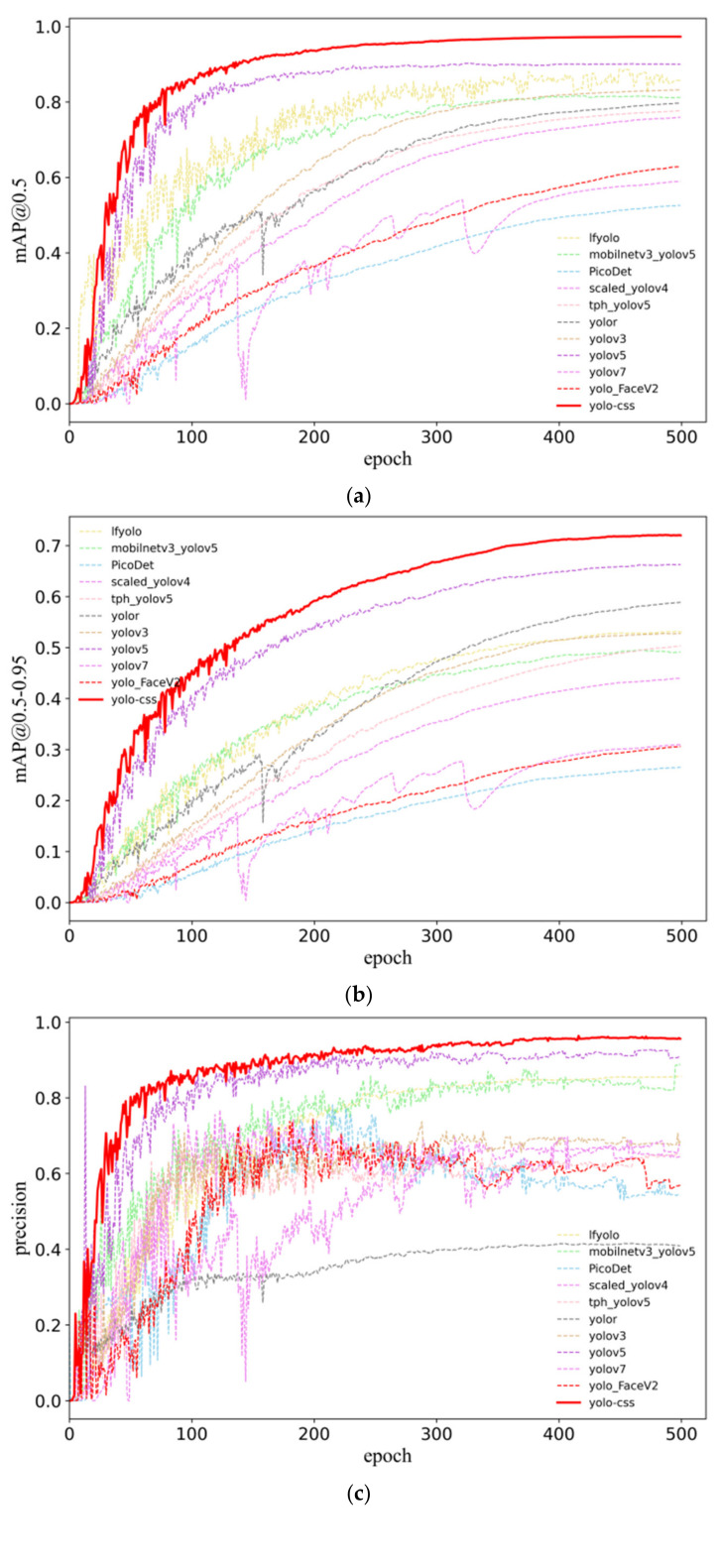

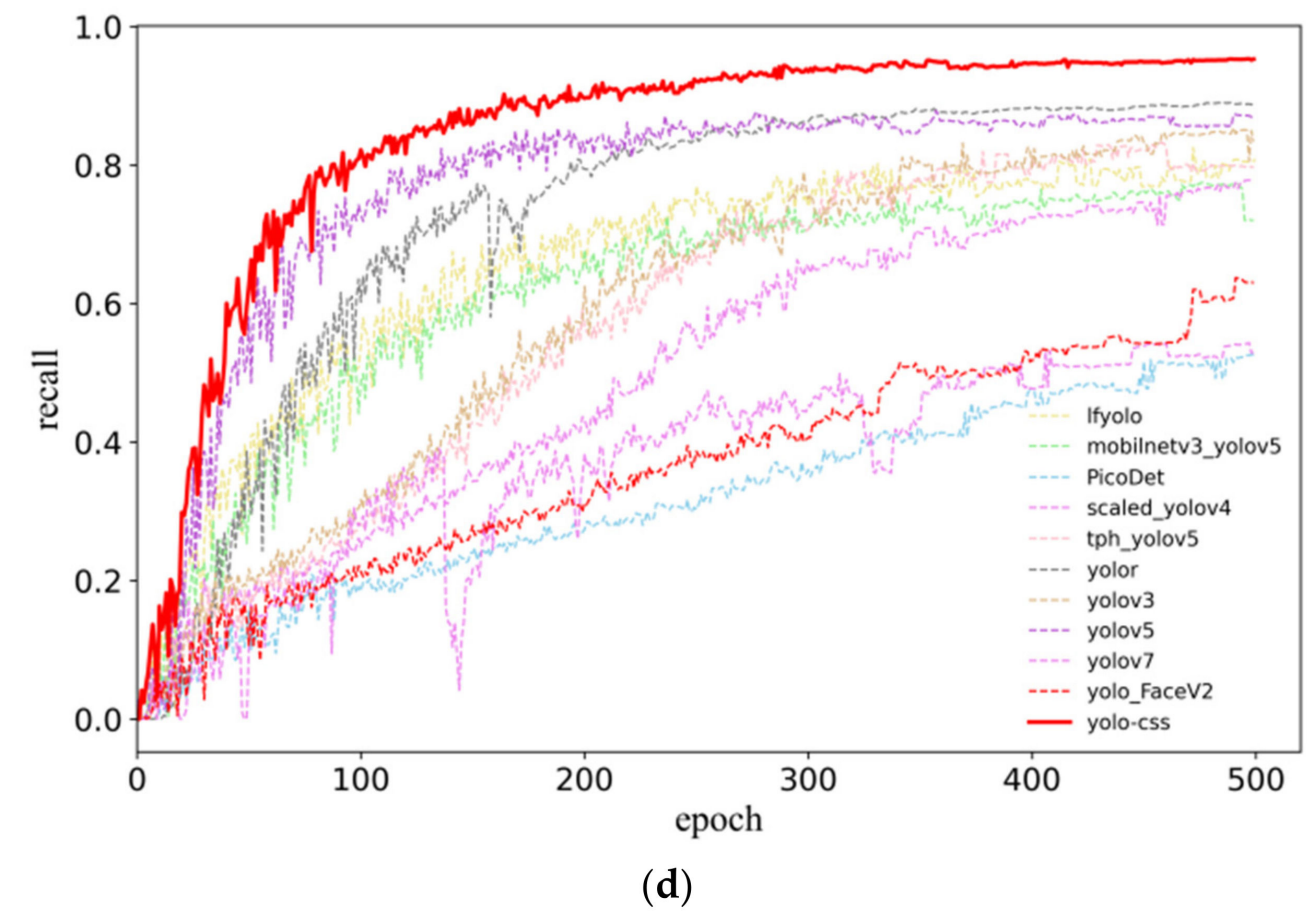
Comparison of YOLO-CSS and other algorithms in terms of mAP 0.5, mAP 0.5–0.95, precision, and recall. (**a**) Comparison of different algorithms in terms of mAP 0.5; (**b**) Comparison of different algorithms in terms of mAP 0.5–0.95; (**c**) Comparison of different algorithms in terms of Precision; (**d**) Comparison of different algorithms in terms of recall.

As shown in Figure 7, the performance trends of mAP 0.5, mAP 0.5–0.95, precision, and recall during the training process are depicted. From the figure, it can be observed that YOLO-CSS (represented by the red curve) exhibits faster convergence and overall smaller fluctuations, indicating its stability. Given that the detected lead bonding defects only occupy a small portion of the entire image, this suggests that our method is more accurate in extracting features for small objects and is more suitable for small object detection. In Figure 7c, it can be observed that the accuracy of YOLO-FaceV2 reaches its peak at approximately 180 epochs and gradually decreases thereafter. Yolor’s accuracy remains around 40% and does not show an increasing trend after 100 epochs. On the other hand, our method demonstrates consistent performance, experiencing a rapid increase in accuracy before the first 100 epochs and then maintaining a slight upward trend, eventually converging at around 95%. This further analysis indicates that our method outperforms the other algorithms.

To further validate the excellent generalization performance of our proposed method, we selected 1241 noisy chip wire bonding X-ray images for detection and compared them with yolov5. The specific test results are shown in Table 3. From the data in the table, it can be seen that due to the presence of noise, the detection accuracy of YOLOv5 is slightly lower than that in Table 2. Under the same conditions, the method proposed in this paper performs better in detecting each type of defect than Yolov5. Therefore, it is sufficient to indicate that the proposed method still has advantages in detection accuracy compared to yolov5 for X-ray images with noise.

**Figure 8:**
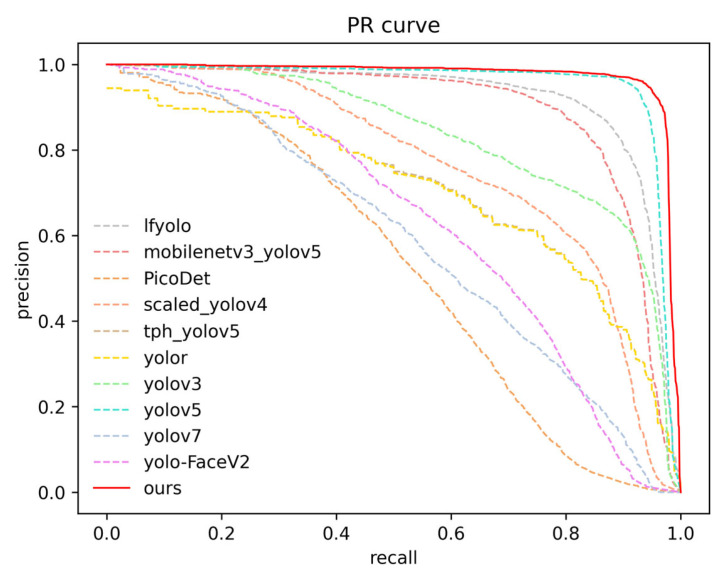
P–R curves of different algorithms.

The P–R curve of the algorithm provides a better reflection of the relationship between precision and recall. A larger area under the P–R curve indicates better performance as the curve is closer to the top right corner. In Figure 8, our algorithm (represented by the red curve) is the closest to the top right corner among all the algorithms, further demonstrating its excellent performance.

**Figure 9:**
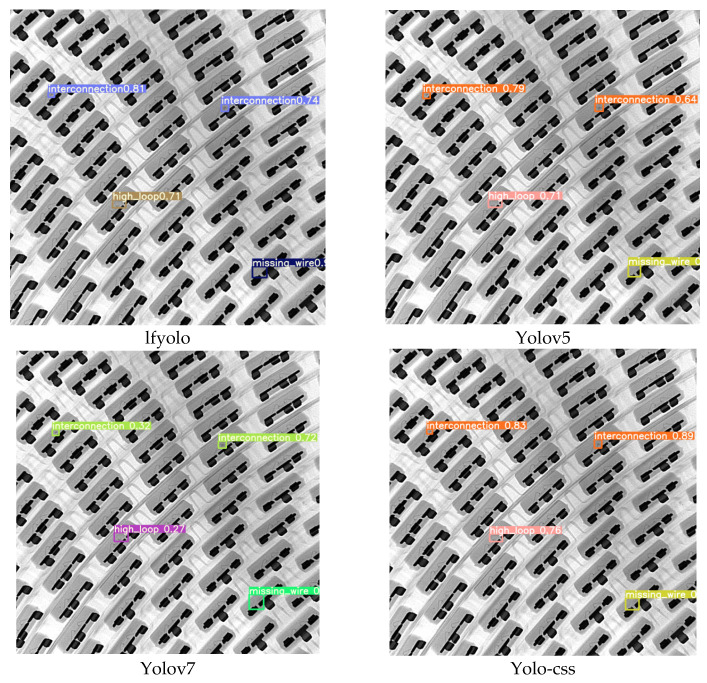
Comparison of detection results of different algorithms.

Partial detection visualizations are shown in Figure 9, comparing the detections of lfyolo, yolov5, yolov7, and yolo-css algorithms. Each algorithm detects different types of defects and provides confidence scores at the end of each defect type. From the analysis of the detection results, it can be observed that our algorithm achieves the best detection performance. Although all four detection algorithms correctly detect the defects, there are noticeable differences in the confidence scores assigned to the detection results. For the two “Interconnection” defects detected by lfyolo, the confidence scores are 0.81 and 0.74, while yolov5 has scores of 0.79 and 0.64. Yolov7 only achieves scores of 0.32 and 0.72. In contrast, our proposed method achieves scores of 0.83 and 0.89, further demonstrating the superiority of our approach. Based on the analysis and comparisons, we can conclude that our method, yolo-css, achieves the highest detection accuracy and recall in the detection of wire bonding defects. With a detection speed of 125 FPS, it strikes a balance between detection accuracy and speed, making it practical for the detection of wire bonding defects in X-ray images.

## 5. Conclusions

This paper proposes a wire bonding defect detection algorithm called YOLO-CSS for X-ray images. We designed the C2SF feature extraction module based on the C2F module structure in YOLOV8, which serves as the backbone of the feature extraction network. Additionally, we introduced a novel adaptive weighted multi-scale feature fusion module (SMA) that adaptively weights the contribution of feature maps from different scales to the detection results. The SMA module is integrated into the neck layer of the model, effectively utilizing the feature information to enhance the feature extraction capability of the network. Furthermore, we employed “skip connections” in the neck layer to effectively utilize the information between feature maps from different layers, improving the model’s ability to capture small objects and enhancing the detection accuracy. Compared to other mainstream algorithms, our method, YOLO-CSS, achieved the highest detection accuracy and recall rate, with mAP, precision, recall, and detection speed reaching 97.3%, 95.7%, 95.2%, and 125 FPS, respectively. These results demonstrate that our YOLO-CSS method effectively balances detection accuracy and speed with a compact model size.

In future research, we will continue to improve the algorithm to enhance its generalization and robustness, further improving its detection performance for application in other small object detection tasks. We will focus on the following directions for future investigation:

- We will continue to explore deep-learning-based object detection algorithms for few-shot learning, aiming to overcome the challenges in acquiring chip wire bonding defect images in practical production scenarios.
- To address the issue of image blurring and indistinct defects caused by low-dose image reconstruction algorithms, we will combine image preprocessing techniques with the yolo-css detection model to improve the accuracy of defect localization and recognition.
- Enhance the algorithm’s generalization capability to detect various types of chip wire bonding defects, thereby reducing production costs in real-world scenarios.

## Figures and Tables

**Table 1:** Comparison of results from different attention mechanisms.

Attention	mAP 0.5 (%)	mAP 0.5–0.95 (%)	Precision (%)	Recall (%)	FPS
None	95.0	68.0	90.6	91.3	139
SENet	96.2	70.6	95.1	94.1	128
ECANet	97.3	71.4	96.1	93.8	130
CA	97.1	70.4	95.7	93.3	131
CBAM	97.3	72.1	95.7	95.2	128

**Table 2:** Comparison of training results on the lead defect dataset among different detection algorithms.

Algorithm	mAP 0.5 (%)	mAP 0.5–0.95 (%)	Precision (%)	Recall (%)	FPS	Size (MB)
LFYOLO [28]	85.7	53.1	85.7	80.6	65.6	14.9
Yolov5-mobilenetv3	81.5	49.5	83.1	76.4	129.8	14.0
PicoDet	52.6	26.5	54.4	52.6	110.2	2.98
Scaled_yolov4 [29]	75.9	43.9	64.3	77.9	71.4	100.0
TPH-yolov5 [30]	77.7	50.3	65.1	79.7	5.7	83.7
Yolor [31]	79.8	58.9	40.9	88.7	87.6	285.1
Yolov3 [32]	83.2	52.8	70.6	80.7	62.5	117.2
Yolov5	90.0	66.3	90.6	87.1	134.1	13.8
Yolov7 [33]	59.0	31.0	67.5	52.9	20.3	71.3
Yolo_facev2 [34]	62.8	30.6	56.7	63.0	131.6	16.6
Ours	97.3	72.1	95.7	95.2	125.9	10.3

**Table 3:** Comparison of detection accuracy between YOLOv5 and our method on noisy data.

Algorithm	High Loop	Low Loop	Interconnect	Broken Wire	Wire Missing
Yolov5	88.1%	91.7%	89.0%	89.1%	93.1%
Ours	93.3%	96.2%	93.6%	96.1%	97.0%

## Data Availability

Data are available on reasonable request from the corresponding authors.
